# New is not always costly: evidence from online processing of topic and contrast in Japanese

**DOI:** 10.3389/fpsyg.2013.00363

**Published:** 2013-06-28

**Authors:** Luming Wang, Petra B. Schumacher

**Affiliations:** ^1^Department of English and Linguistics, Independent Emmy Noether Research Group, Johannes Gutenberg University of MainzMainz, Germany; ^2^Department of Germanic Linguistics, Philipps University of MarburgMarburg, Germany

**Keywords:** Japanese, topic, contrast, exhaustivity, expectation, updating, N400, Late Positivity

## Abstract

Two visual ERP experiments were conducted to investigate topic and contrast assigned by various cues such as discourse context, sentential position, and marker during referential processing in Japanese. Experiment 1 showed that there was no N400-difference for new vs. given noun phrases (NPs) when the new NP was expected (contrastively focused) based on its preceding context and sentential position. Experiment 2 further revealed that the N400 for new NPs can be modulated by the NP's contrastive meaning (exhaustivity) induced from the marker. Both experiments also showed that new NPs engendered an increased Late Positivity. The reduced N400 for new vs. given supports an expectation-based linking mechanism. In addition, costs that were consistently observed for new vs. given entities emerged in a subsequent process, in which the new NP's occurrence requires updating and correcting of the discourse representation built so far, which is indexed by an enhanced Late Positivity. We argue that the overall data pattern should be best explained within a multi-stream model of discourse processing.

## Introduction

In order to study language processing in a natural environment like everyday communication, recent neurophysiological research has shown increasing interest in the influence of context during word or sentence processing^1^. Crucially, contextual influence can be observed beyond the sentence-level. For instance, when sentences are processed as part of a continuous text, the discourse function (e.g., topic, focus), and information status (given vs. new) of a referential expression is computed and this constrains the way in which one refers back to this referent subsequently. This is evident from the so-called *Repeated Name Penalty*. Following a sentence such as *Bruno was the bully of the neighborhood*, a subsequent sentence using the name again (*Bruno*) leads to increased processing costs compared to a pronominal counterpart (*he*) (see behavioral findings in Gordon et al., 1993 and neurophysiological findings in Streb et al., 1999). This preference for a pronoun emerges immediately as the result of the two sentences being combined to form a coherent discourse (see Gernsbacher, 1997; Kehler, 2002 on discourse coherence). The preference as such cannot be explained by sentence-level processing in a straightforward way as it requires a broader notion of context to account for intersentential relations and anaphoric chains. Thus, a discourse model that takes into account the discourse/information packaging function of the context is needed to explain referential processing in these cases. In addition to context, other factors such as morphological markers or word order contribute to information packaging. Here, we investigate discourse processing in Japanese, a language that utilizes context, positional information, and morphological marking to indicate discourse functions.

The work presented here starts from referential processing captured within a discourse model, which takes context as a discourse-level phenomenon (see also van Berkum et al., 1999). Context has predictive potential in how the next sentence packages the information (e.g., topic-comment; background-focus). For example, a contextually given referent qualifies as topic of the next sentence and topic-continuity is preferred over topic-shift (cf. Gordon et al., 1993). Crucially, discourse context has its own structure and representation and uses discourse functional information in building up a coherent representation. Discourse representation structure is distinct from syntactic representation in covering intersentential and textual relations, encoding transitional states between utterances, and so on. Furthermore, and of central concern in the present study, morphosyntactic cues also bear information structural function. For instance, different referential forms [full noun phrases (NPs) or pronouns, definite or indefinite NPs] correspond to discrete information status used to encode contextually given referents or to introduce new referents (e.g., de Villiers, 1974; Gundel et al., 1993; Gernsbacher and Robertson, 2002). Similarly, sentential position conveys discourse functionality as well, such as the correspondence between sentence-initial position and topicality (cf. Gundel, 1988). A model of referential processing must therefore be capable of capturing the correspondences between morphosyntactic instantiations and their discourse functions. Such a syntax-discourse interface view allows us to investigate the complex system of referential processing in which multiple morphosyntactic cues contribute to dynamically construct and update discourse representation.

In the field of referential processing, it is commonly observed that contextually new NPs engender processing cost in comparison with given NPs (cf. e.g., Clark and Haviland, 1977; Yekovich and Walker, 1978; Arnold et al., 2000). In the following, we want to test whether this disadvantage for new information is attributable to the information status *per se* or can be linked to a more general capability of the human brain, namely expectation-based parsing. In particular, we adopt a discourse functional perspective, whereas topical entities are preferably given (Givón, 1983; Gordon et al., 1993). According to the expectation-based account, the parser privileges given information for topical entities (“expecting given”). To demonstrate the validity of expectation-based parsing, however, it is better to look at cases in which the discourse context induces the expectation of an upcoming new NP (“expecting new”). For instance, a new NP may represent contrastively focused information according to its preceding context. If expectation matters, we will observe reduced processing cost for an expected new NP during contrast processing.

The present investigation thus compares topic and contrast processing and examines whether the processing disadvantage for a new NP can be reduced based on context and morphosyntactic cues, utilizing event-related brain potential (ERP) measures. We test this in Japanese because this language offers rich morphosyntactic cues bearing discourse functions. It does not only have sentential position as a cue to encode topic and contrast like previously examined languages, but also has the discourse marker *wa* to encode these discourse functions. Furthermore, case markers in this language also meet discourse requirements (e.g., the nominative case marker *ga* can mark exhausitive contrast instead of merely indicating subjecthood). In the following subsections, we first review previous ERP studies of topic and contrast processing, with an introduction to the Syntax-Discourse Model (SDM) that accounts for aspects of information packaging. Then we provide a brief outline of the theoretical background of topic and contrast in Japanese, with a focus on the discourse functions of sentential position and markers in this language. Subsequently, we present the specific predictions for the two ERP studies on discourse context, sentential position, and markers in Japanese. Experiment 1 manipulated sentential position (NP1 vs. NP2) and discourse marker (with vs. without *wa*) of a dative object following three types of discourse contexts (Given vs. Inferred vs. New). Experiment 2 manipulated the three markers (*ga*, *o*, *wa*) for the initial NP following the same three discourse contexts.

### Referential processing in the SDM

Research on the comprehension of referential expressions has investigated the role of different information status (i.e., degrees of givenness), the information structural contributions of topic and focus, and their interaction with syntax and prosody. In addition to a benefit of given over new information in terms of processing load, research revealed a given-before-new ordering preference as well as a general form-function correlation (e.g., Clark and Haviland, 1977; Bock and Irwin, 1980; Almor, 1999; Arnold et al., 2000; Carlson et al., 2009). As far as topicality is concerned, topic-continuity is preferred over topic-shift (Gordon et al., 1993; Hung and Schumacher, 2012). Research also suggests that topical and focused entities raise the cognitive salience of their referents (Almor, 1999; Cowles et al., 2007). Topic and corrective focus have further been shown to be capable of overriding syntactic preferences (Kaiser and Trueswell, 2004; Bornkessel and Schlesewsky, 2006). To provide a solid basis for our investigation, we now concentrate on ERP findings from referential resolution and present a dynamic model of discourse processing.

Previous ERP studies have shown that there is a robust influence of the discourse context on referential processing. For example, Burkhardt (2006) compared ERP responses to an NP such as *the conductor* in the sentence *He said that the conductor was very impressive*, which followed three different types of discourse context (in the following, English translations are given of the original German materials): (a) Given context: *Tobias visited a conductor in Berlin*; (b) Inferred context: *Tobias visited a concert in Berlin*; (c) New context: *Tobias talked to Nina*. The findings revealed a graded N400 as a function of contextual fit (N400: New > Inferred > Given) and a subsequent Late Positivity following the Inferred and New context (Late Positivity: Inferred/New > Given). The data pattern suggested that two core mechanisms are engaged in referential processing, i.e., *Discourse Linking* and *Discourse Updating*, as captured within the SDM. Notably, the two processes are independent from each other. This is evidenced by the observation that some referential expressions evoke a biphasic pattern (e.g., given vs. inferred entities in sentence-medial position; Burkhardt, 2006), some only an N400 difference (e.g., given vs. inferred entities in sentence-initial position in German; Schumacher and Hung, 2012) and others only a Late Positivity difference (e.g., inferred entities representing necessary vs. probable instruments; Burkhardt, 2007).

Regarding the first mechanism in the SDM, incoming information is linked to previously established discourse. This process is modulated by the parser's anticipation of an upcoming word, which is not just a function of the lexical-semantic distance between the word and the potential anchor expression in discourse (cf. e.g., Federmeier and Kutas, 1999), but is also contingent on extra-lexical factors such as co-textual expectations (van Berkum et al., 1999) or discourse salience (e.g., topicality in Hung and Schumacher, 2012), and prosodic cues (Heim and Alter, 2006; Toepel et al., 2009; Schumacher and Baumann, 2010; Baumann and Schumacher, 2012). As such, this process represents the attempt of connecting to what has been uttered before in a coherent manner. If the most anticipated expression is encountered, linking attempts are cheap; if the upcoming referential expression deviates from the expected one on a variety of factors, processing demands accrue, resulting in a more pronounced N400. Crucially, the nature of the N400 has been subject to much debate. It has been associated with expectation (Kutas and Hillyard, 1980), lexical activation (Federmeier and Kutas, 1999), or postlexical integration (Brown and Hagoort, 1993). In the following, we explore expectation-based parsing, namely the reduction of processing cost for an expected new NP and the question which cues affect the generation of expectations.

The *Discourse Updating* process reflected in a Late Positive potential reveals costs from adding new discourse referents (cf. Burkhardt, 2006; Kaan et al., 2007; Hirotani and Schumacher, 2011), modifying previously introduced discourse representation structure (cf. Burkhardt, 2007), and shifting to a new topic (cf. Hung and Schumacher, 2012). Focus also evokes a positive deflection (Bornkessel et al., 2003; Bornkessel and Schlesewsky, 2006; Cowles et al., 2007; Stolterfoht et al., 2007) as well as updating triggered by violations of exhaustivity (Drenhaus et al., 2011). What these cases have in common is that they represent discourse-internal reorganization and appear to reflect most directly mapping operations between syntax and discourse. One of these mappings is the correspondence between an NP in syntax and a corresponding discourse representation. Another mapping operation is tied to the functional contribution of sentential position, e.g., the correspondence between a sentence-initial entity and its role as aboutness-topic in discourse.

Initial investigations of the impact of discourse markers in German indicate that *Discourse Linking* processes appear to be computed independent from the choice of discourse marker. Schumacher (2009) manipulated the definiteness of the critical NP in the target sentence (in German), i.e., *a conductor* vs. *the conductor*, following the three types of discourse contexts outlined above (Burkhardt, 2006). ERP responses time-locked to the head noun revealed the same contextually modulated N400 observed for both definite and indefinite NPs (New > Inferred > Given). But in contrast to the definite NPs, there was a Late Positivity for all indefinite NPs (relative to the Given definite NP). The results suggested that definiteness marking does not influence *Discourse Linking* in German, but is considered during the *Discourse Updating* stage, where a new discourse representation must be introduced for the respective NP.

Though definiteness marking is not available in Japanese, the given-new distinction can be realized by the distinctive usage between a (topic) marker *wa* and a (subject) marker *ga* in this language. Hirotani and Schumacher (2011) conducted a Japanese experiment similar to the German study presented above (Burkhardt, 2006) with the exception that they manipulated the *wa/ga* marker at the critical (subject) NP. This manipulation was based on the notion that a nominative case-marked subject is typically contextually new (NP-*ga*) while a topic-marked entity should be given (cf. Kuno, 1973). The experimental design from Hirotani and Schumacher (2011) is illustrated in (1). The critical NP, either marked with *ga* or *wa*, is underlined.


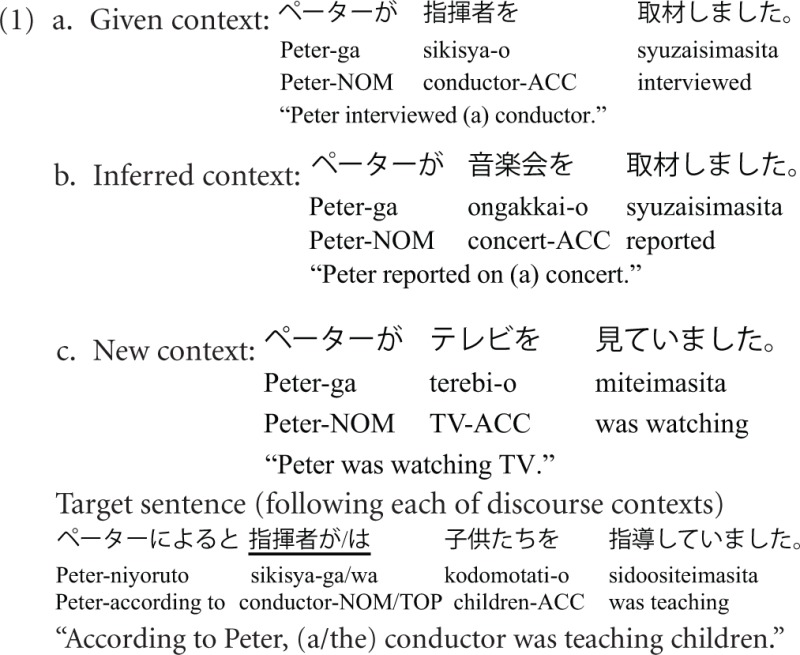


The results confirmed the findings from the German study on definiteness markers. There was a context-induced N400 (New > Inferred > Given) irrespective of the NP's marker. However, a Late Positivity was observed for a process that could be described as topic-shift, i.e., when *wa*-marked NPs followed discourse contexts in which they were not established as a topic yet, but licensed by a particular semantic set relation (NP-*wa* in the Inferred context). Again, markers were observed to influence *Discourse Updating* rather than *Discourse Linking*.

The findings from German and Japanese revealed an overwhelming power of discourse context over markedness in assigning information status to an NP. This is most evident by the fact that in both studies the contextually new NP engenders linking cost even though the local marker indexes a Given reading. However, there are a few open questions which we seek to address in the present research: (i) Both studies found increased linking costs for new NPs in topic processing, where costs could be accounted by topicality (a new NP is not a good topic), or accounted by expectation (a new NP is less expected to be a topic). Yet, what will happen if a new NP is not a topic but still predictable from context? Instead of using a New context like (1c), we use a New context that biases the NP toward a contrastive focus reading. (ii) The two studies manipulated markers at the grammatical subject of the target sentence. Thus, it is difficult to disentangle the Given/topic-preference from a subject-first preference or a sentence-initial preference (“given = topic = subject = sentence-initial”). In order to minimize the influence of this overlap, we target the dative object rather than the subject because the former shows less overlap with a particular discourse function or sentence position. (iii) *Wa* and *ga* have more discourse functions than what has been tested so far. Applying Kuno's classification to the stimuli in (1), *wa* following the Given context (1a) is topical/non-contrastive, and *ga* following the New context (1c) is descriptive, because it indicates that the speaker gives a neutral description. However, as discussed in the next section, *wa* and *ga* also convey contrastive function. Unless contrast processing is tested, the finding that these markers do not influence *Discourse Linking* should be restricted to topic processing. Before we move to the experiments, the discourse functions of sentential position and markers in Japanese need to be detailed.

### Topic and contrast in japanese

An essential dimension of information structure is topic, which corresponds to an entity that represents what the rest of the utterance is about. With this definition, we follow Reinhart (1981) and similar accounts that assume that a certain expression is used as an address or starting point for subsequent information storage, thereby representing a salient unit for mental organization. In addition, topic is widely observed to be constrained by the givenness of the respective entity in discourse context (except a contrastive topic). Topic contrasts with focus, which (implicitly) evokes the presence of a set of alternatives and is often viewed as an answer to a *wh*-question (Rooth, 1992). Another information structural dimension that is relevant for the present discussion is contrast, which explicitly indicates an alternative and draws from a more restricted alternative set (cf. Repp, 2010). Unlike topic, contrast can be a contextually given or new entity. Cross-linguistic research indicates that contrast can fall together with both topic and focus and should therefore be considered an independent dimension of information structure (cf. *contrastive topic* and *contrastive focus* in Büring, 1997; Hara, 2006; Heycock, 2008; Neeleman et al., 2009; Tomioka, 2010; Vermeulen, 2011; among others). Consider “*What did Nick have for dinner?—Well, TIM had pasta.”* where *Tim* represents a contrastive topic resulting from the overlap between the topic and contrast dimensions. On the one hand, it represents topic and sets up what the sentence is going to be about, and on the other hand, it implicitly evokes the presence of a set of alternatives.

When characterizing Japanese, it turns out that both topic and contrast can be realized by the same marker (*wa*). The topical *wa* and a contrastive *wa* can be distinguished by sentential position, by discourse context, or by both of them, as observed by Kuno (1973, p.38)^2^. Recently, some accounts have demonstrated a stricter mapping between *wa*'s discourse function and sentential position. Topic has been argued to occur in sentence-initial position, while contrast, in turn, may occur sentence-initially and -medially (cf. e.g., Heycock, 2008; Neeleman et al., 2009; Vermeulen, 2011, 2013). As far as contrastive topic and contrastive focus are concerned, Vermeulen (2010) demonstrates that—like aboutness-topic—contrastive topic must appear at the sentence-initial position, above the position of contrastive focus^3^. In this way, an initial *wa*-marked entity maps onto a topic, while a non-initial *wa*-marked entity maps onto a contrast but not topic, e.g., a contrastive focus. It is then the sentence-initial position rather than the *wa* marker itself that licenses a topic in this language (Hara, 2006; Tomioka, 2007; Neeleman et al., 2009). The two position-dependent functions of the *wa* marker will be examined in Experiment 1.

Another important observation arising from Kuno's work is that the use of the *ga*-marker is not restricted to just marking a subject but conveys a discourse function in the presence of a discourse context. Kuno (1973) separates a descriptive *ga* from an exhaustive listing *ga*. Whereas the descriptive *ga* marks an informatively new referent, the exhaustive *ga* can further be understood to mark a contrast, in that it represents the exclusion of all other alternatives (in this case *Kyoko-ga* implies “Kyoko and only Kyoko”). The exhaustive contrast reading of the *ga* marker is supported by a corpus analysis of *ga* in Japanese conversational discourse (Ono et al., 2000). The present study takes the same view by treating *ga* as a discourse marker rather than a case marker (in analogy to *wa*) in Experiment 2.

Up to now, research has focused on the more well-known distinction between topical *wa*-marked NPs and descriptive *ga*-marked NPs (see Hirotani and Schumacher, 2011), similar to the definiteness distinction observed in English and German. Yet, to obtain a clearer picture of the markers' discourse function, an investigation of contrast processing is also needed. The research on referential processing reviewed above either investigated topic processing alone or manipulated discourse context in combination with sentential position or markedness separately. In Japanese, topic has been associated with either marker (topical *wa*) or position (NP1) and contrast corresponds with either marker (contrastive *wa*, exhaustive *ga*) or position (here NP2). These features of Japanese offer an excellent opportunity to investigate the intricate system of topic and contrast processing in a language that appears to employ multiple cues.

## Topic and contrast (Experiment 1)

In the present study, we directly compare topic and contrast processing in a contextually licensed situation to compare “expecting given” vs. “expecting new,” respectively. We utilized a context that induces a contrastive reading of a new NP by inserting a negation at the beginning of the target sentence (e.g., *Mr. Satoo returned the record to the director, didn't he?—No…”*) (cf. previous research on contextually-induced contrastive reading by Bornkessel and Schlesewsky, 2006; Cowles et al., 2007). In this way, the NP [e.g., *(to) the librarian*] is lexically new as in previous studies, but its occurrence is expected after the negation (inducing corrective contrast), which is different from the previous studies where the new NP has been introduced out of the blue. This condition was compared to a given and an inferred context. Besides context manipulations, we also manipulated the sentential position of the critical NP (NP1 or NP2) and *wa* marker (with or without *wa*), since these cues may contribute to topicality and contrastiveness as well.

The sample stimuli are presented in Table 1. In Japanese, *wa*-marking of a dative object can be distinguished from a subject or an accusative object with respect to its form. When the subject or the accusative object is used as a topic or contrastively, the nominative and accusative case marker is obligatorily replaced by *wa* (NP-*wa*); by contrast, a topical or contrastive dative object usually maintains its dative case marker *ni* (NP-*ni*-*wa*)^4^. This allowed us to minimize grammatical function ambiguity at the critical NP. More importantly, unlike subjects, dative objects are less biased toward the topical reading.

**Table 1:**
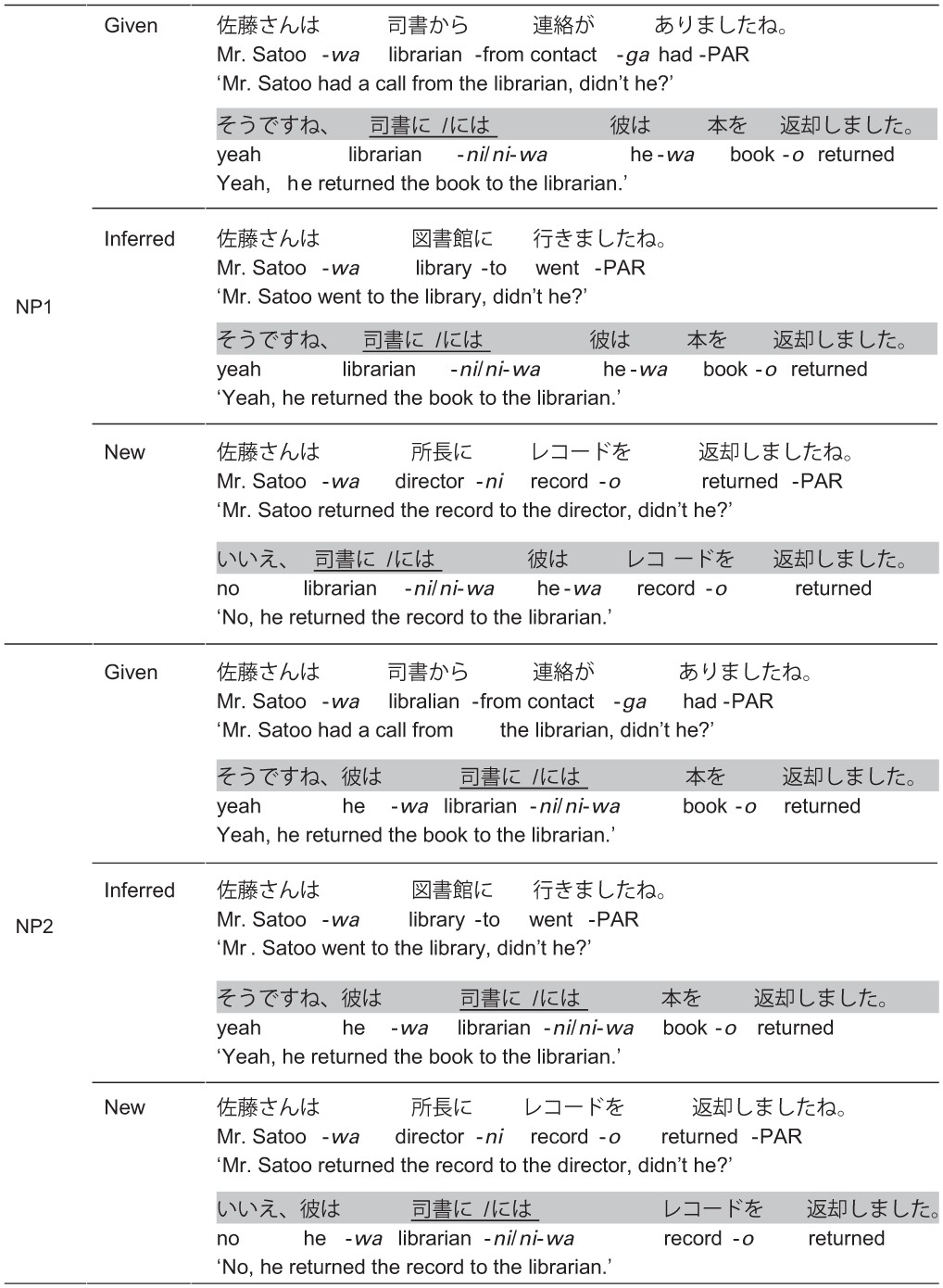
**Examples of critical conditions in Experiment 1 for the factors position (NP1, NP2), marker (*ni*,* ni-wa*), and context (Given, Inferred, Contrastive New)**.

*Target sentences are shown with gray background and the critical dative objects are underlined*.

The contrastive New condition is the critical condition to test the prediction that new information is expected under certain circumstances. The Given and Inferred conditions are used as control conditions, representing topic processing as examined so far. According to the SDM and expectation-based accounts, the N400 provides an indication of expectedness computed on the basis of cue availability and strength during topic and contrast processing. For topic processing at the NP1 position—given this position is closely tied to topic no matter it is contrastive or not—we expect to replicate previous N400-differences, being most pronounced for new NPs, less pronounced for inferred NPs, and most reduced for given NPs (i.e., New > Inferred > Given). The critical question is whether the N400-amplitude reduces for expected new NPs. This is examined through contrast processing, especially at the NP2 position, where a contrastive focus reading is guaranteed (cf. Vermeulen, 2010). The account of expectation-based parsing predicts a similarly reduced N400 for New vs. Given in this case because the new NP fulfills the expectation of “new = contrastive focus” generated by contextual and positional cues (perhaps also *wa* marker). Alternatively, if the processing disadvantage for new NPs arises from the information status *per se*, then we should still observe a pronounced N400 for new NPs independent of discourse context and other cues.

In addition, since *Discourse Updating* costs accrue when a new discourse unit must be introduced or previous discourse structure must be modified, we expect main effects of discourse context as observed in previous research (New/Inferred > Given). Although new NPs—no matter whether they are informatively new as in previous research or contrastively new as in the present study—cause the necessity of updating the discourse structure, we assume that contrastive new NPs require more updating effort, because in addition to introducing a new entity into discourse structure, they call for correction of previously established discourse structure. Hence, we should observe a more pronounced Late Positivity for the New condition regardless of the NP's position and markedness.

### Methods

#### Participants

Twenty-seven monolingually raised native speakers of Japanese participated in the experiment after giving informed consent (20 women; mean age: 25.1 years; range: 19–40 years). At the time of the experiment, all participants were residing in Germany. Participants were right handed (as assessed by an adapted Japanese version of the Edinburgh handedness inventory; Oldfield, 1971) and had normal or corrected-to-normal vision. Three participants were subsequently excluded from the final data analysis on the basis of excessive EEG artifacts and/or too many errors in the behavioral control task.

#### Materials

Table 1 illustrates 12 conditions examined in Experiment 1. Each of the sentences contained three nouns and a dative verb [or compound dative verb such as *yon-de-agemashita*, “read (something) for (someone)”] in a string of NP1-NP2-NP3-Verb. The total number of characters for each critical dative NP was held constant across conditions: only two-character nouns were used for dative NPs. Forty sets of the 12 conditions were constructed. In order to ensure that the experimental sentence pairs did not only meet our experimental constraints, but were also kept as natural as possible, we first conducted an acceptability rating study, using materials reflecting the structures illustrated in Table 1. The details of this pretest are reported in the Appendix. With this acceptability rating, we also wanted to determine the best marking of the subject NPs in the experimental stimuli. On the basis of the acceptability results, we chose the *wa*-marker for all subject NPs in Experiment 1.

The 480 trials (40 in each condition) were interspersed with 240 filler trials. For Given and Inferred contexts, we included transitive and intransitive sentences. By using fillers starting with *yes* and ending with various verbs, it is unlikely for participants to predict a particular target sentence after “
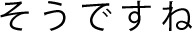
” (*yeah*). For New contexts, we included transitive sentences with different NPs or verbs because one can negate the object of the event but also the event itself. Overall, the fillers ensured a variety of sentence types. The 720 trials were presented to participants in two different randomized presentation orders. In order to ensure that the dative object could receive an unambiguously recipient reading in the described action, dative verbs that take a complement clause such as *introduce* were excluded from the present study. Due to the length of the experiment, the whole experiment was separated into two sessions, which were separated by a time interval of at least 2 weeks. Statistical analyses registered no session effects.

#### Procedure

The experiment was conducted in a dimly lit, sound attenuated room. Participants were seated ~1.2 m in front of a 17-inch computer screen. Each session began with a short training session followed by eight experimental blocks. Each block comprised 45 trials. Participants took short breaks between blocks. Each block lasted ~8 min.

Trials were presented visually in the center of a computer screen. The context sentences were presented as a whole (no space between words) with a presentation time of 2500 ms and the target sentences were presented in a word-by-word manner with a presentation time of 650 ms per word. Each trial began with the presentation of an asterisk (600 ms stimulus onset asynchrony; SOA) and ended with a 1500 ms pause. Subsequently, participants were required to complete a comprehension task by answering a yes/no question based on the content of the preceding context or target sentences.

Comprehension questions to be answered with *yes* (50% of all questions) were consistent with the proposition of the preceding sentence. Questions to be answered with *no* included a substituted subject, object, or verb. Comprehension questions were presented on the screen as a whole with a question particle “
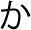
” at the end. The comprehension task required the answer *yes* equally often as *no* in each of the experimental conditions.

The assignment of the left and right buttons to the answers for the comprehension task was counterbalanced across participants. Participants were asked to avoid movements and blinks during the presentation of the target sentences.

#### EEG recording

**Figure 1:**
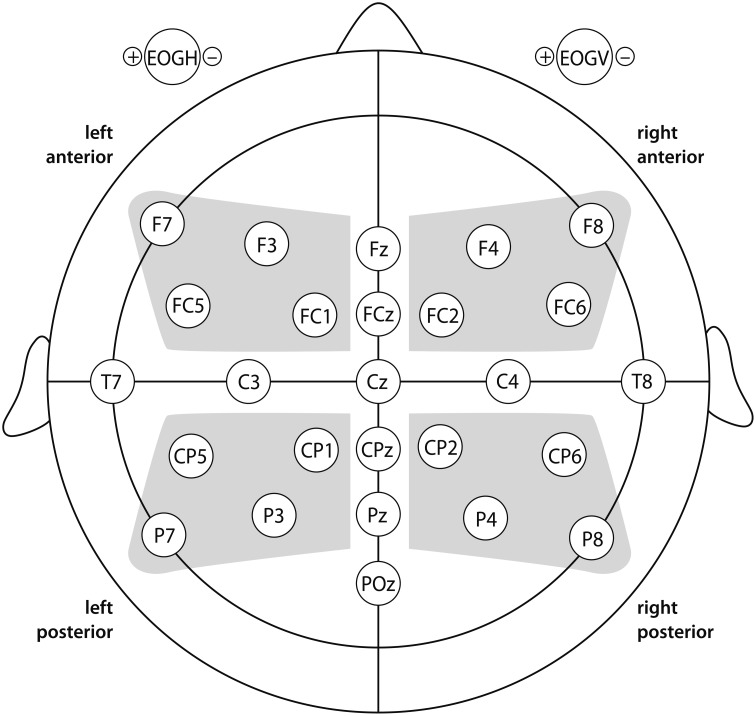
**A top view of the scalp (up = forward; left = left)**. Additional electrodes labeled as “EOGH” and “EOGV” refer to the electrodes that record the horizontal and vertical electrooculogram. Statistical analysis involved the topographical factor “region of interest” (ROI). Lateral regions of interest are indicated by shaded areas: left-anterior (F3/F7/FC1/FC5); left-posterior (CP1/CP5/P3/P7); right-anterior (F4/F8/FC2/FC6); and right-posterior (CP2/CP6/P4/P8). For midline sites, each electrode was defined as a ROI of its own (FZ/FCZ/CZ/CPZ/PZ/POZ).

The EEG was recorded via 27 AgAgCl-electrodes (ground: AFZ) fixed at the scalp by means of an elastic cap (*Easycap*, Munich, Germany), as shown in Figure 1. Recordings were referenced to the right mastoid and re-referenced to linked mastoids offline. Electrode impedances were kept below 5 kΩ. All EEG and EOG channels were amplified using a *BrainAmp* DC amplifier (Munich, Germany) and recorded with a digitization rate of 500 Hz. EEG data were filtered with 0.3–20 Hz band pass off-line to exclude slow signal drifts.

Average ERPs were calculated per condition per participant from the onset of the critical stimulus items (i.e., dative object) to 1200 ms post-onset, before grand-averages were computed over all participants. Trials for which the comprehension task was not performed correctly were excluded from the averaging procedure, as were trials containing ocular, amplifier saturation, or other artifacts (EOG rejection criterion: ±40 μV). Less than 13% of all trials were excluded in this manner and exclusion rates did not differ significantly across conditions (*F* < 1).

#### Data analysis

We computed repeated measures ANOVAs involving the three factors: discourse context (CO), Given vs. Inferred vs. New; discourse markedness (MA), with *wa* vs. without *wa*; sentential position (NP), NP1 vs. NP2. ERP responses relative to the dative object were calculated for mean amplitude values per time window per condition. “Region of interest” (ROI) was defined as in Figure 1. Time-windows were chosen on the basis of visual inspection of the data. The statistical analysis was carried out in a hierarchical manner, i.e., only significant effects (*p* < 0.05) were resolved. To avoid excessive type1 errors due to violations of sphericity, we applied the correction of Huynh and Feldt (1970) when the analysis involved factors with more than one degree of freedom in the numerator. Significant effects of CO were followed up by means of Bonferroni-adjusted pair-wise comparisons between the critical conditions.

The time-windows chosen for statistical analysis were first obtained by visual inspection and then verified by a 50 ms interval analysis, whereby analyses were carried out on the basis of intervals of 50 ms length over the range from onset to 900 ms thereafter. The same effects observed in at least two successive windows (≥q 100 ms) were considered stable (cf. Gunter et al., 2000 for details about this procedure). Effects observed only in one 50 ms window, or in several non-adjacent 50 ms window, were considered unstable and not considered for further statistical analysis. In this way, we determined the 350–500 ms window for further analyses. As there were straightforward context effects in our ERP plots between 500 and 700 ms, but an interaction of ROI × NP × MA in a shorter window between 550 and 700 ms, we chose to run separate analyses over these two later windows.

### Results

**Figure 2:**
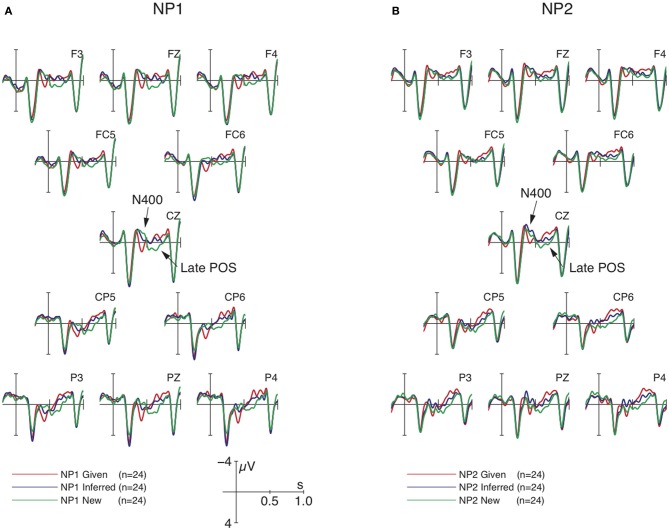
**Grand average ERPs (*n* = 24) time-locked to the dative NP (onset at the vertical bar) in the Given, Inferred, Contrastive New contexts of Experiment 1**. Comparisons of NP1 vs. NP2 are shown in Panels **(A)** and **(B)**, respectively. Negativity is plotted upwards.

ERP responses time-locked at the position of the dative object suggested an overall context-induced biphasic N400-Late Positivity pattern, replicating previous findings from referential processing in German, Chinese, and Japanese. We report statistics for the two time-windows separately. See Table 2 for effects that reached significance. Between 350 and 500 ms, the highest-level statistical analysis revealed an interaction of ROI × NP × CO and an interaction of ROI × MA × CO [lateral: *F*_(6, 138)_ = 2.62, *p* < 0.02; midline: *F*_(10, 230)_ = 1.88, *p* < 0.05]. Resolving these interactions by ROI showed a significant effect of NP × CO in all regions except the left-anterior region (lateral: all *F*s > 4.11, *p*s < 0.002; midline: all *F*s > 6.36, *p*s < 0.004). The interaction of MA × CO did not reach significance in any of the regions. Since there was no markedness effect in the N400-window, we combined *wa*-marked and non-*wa*-marked conditions for analyzing the interaction of position and context. Subsequent repeated-measures ANOVA revealed main effects of CO and NP and support a clear interaction of the critical factors NP × CO, shown in Figure 2. The data pattern observed at NP1 fully replicated previous findings from German and Japanese at the sentence-initial position by showing a graded N400 as a function of context type, i.e., New > Inferred > Given. However, at NP2, this N400 was observed for the comparison of the Inferred context relative to the New/Given context, i.e., Inferred > New/Given.

**Table 2:** **Analysis of variance (ANOVAs) of the mean ERP amplitudes in Experiment 1**.

**N400 (350–500 ms)**					
			***df***	***F***	***p***
**LATERAL REGIONS**					
NP			1.23	16.91	^***^
CO			2.46	18.02	^***^
ROI × NP × CO			6.138	4.58	^**^
ROI = R-ant	NP1	Inferred vs. Given	1.23	18.20	^***^
		New vs. Given	1.23	47.04	^***^
		New vs. Inferred	1.23	10.21	^**^
	NP2	Inferred vs. Given	1.23	7.61	^*^
		New vs. Given	1.23	−	−
		New vs. Inferred	1.23	12.29	^**^
ROI = L-post	NP1	Inferred vs. Given	1.23	19.36	^***^
		New vs. Given	1.23	39.43	^***^
		New vs. Inferred	1.23	4.84	^**^
	NP2	Inferred vs. Given	1.23	7.95	^**^
		New vs. Given	1.23	3.53	·
		New vs. Inferred	1.23	4.06	·
ROI = R-post	NP1	Inferred vs. Given	1.23	28.66	^***^
		New vs. Given	1.23	34.22	^***^
		New vs. Inferred	1.23	−	−
	NP2	Inferred vs. Given	1.23	20.24	^***^
		New vs. Given	1.23	−	−
		New vs. Inferred	1.23	16.72	^***^
**MIDLINE REGIONS**					
NP			1.23	12.53	^**^
CO			2.46	22.39	^***^
ROI × CO			10.230	5.38	^**^
NP × CO			2.46	13.96	^***^
	NP1	Inferred vs. Given	1.23	26.31	^***^
		New vs. Given	1.23	54.83	^***^
		New vs. Inferred	1.23	6.90	^*^
	NP2	Inferred vs. Given	1.23	15.39	^***^
		New vs. Given	1.23	3.90	·
		New vs. Inferred	1.23	10.58	^**^
**Late Positivity (context effect: 500–700 ms)**					
**LATERAL REGIONS**					
NP			1.23	12.29	^**^
CO			2.46	43.95	^***^
ROI × CO			6.138	7.89	^***^
ROI = L-ant		Inferred vs. Given	1.23	2.57	−
		New vs. Given	1.23	20.35	^***^
		New vs. Inferred	1.23	22.11	^***^
ROI = R-ant		Inferred vs. Given	1.23	3.60	−
		New vs. Given	1.23	43.86	^***^
		New vs. Inferred	1.23	34.93	^***^
**Late Positivity (context effect: 500–700 ms)**					
ROI = L-pos		Inferred vs. Given	1.23	14.13	^**^
		New vs. Given	1.23	66.56	^***^
		New vs. Inferred	1.23	52.95	^***^
ROI = R-pos		Inferred vs. Given	1.23	10.05	^**^
		New vs. Given	1.23	69.38	^***^
		New vs. Inferred	1.23	59.90	^***^
**MIDLINE REGIONS**					
NP			1.23	4.45	^*^
CO			2.46	45.89	^***^
		Inferred vs. Given	1.23	7.77	^*^
		New vs. Given	1.23	65.99	^***^
		New vs. Inferred	1.23	66.32	^***^
**Late Positivity (markedness effect: 550–700 ms)**					
**LATERAL REGIONS**					
ROI × NP × MA			3.69	8.33	^***^
ROI = L-ant	NP2		1.23	9.2	^**^
ROI = R-ant	NP2		1.23	19.52	^***^

Note: L-ant, left anterior region; L-post, left posterior regions; R-ant, right anterior region; R-post, right posterior regions. NP1, sentence-initial position; NP2, sentence-medial position. – no significance; · p < 0.08;

* p < 0.05;

** p< 0.01;

*** *p < 0.001*.

*Interactions are resolved by the factor from the left*.

**Figure 3:**
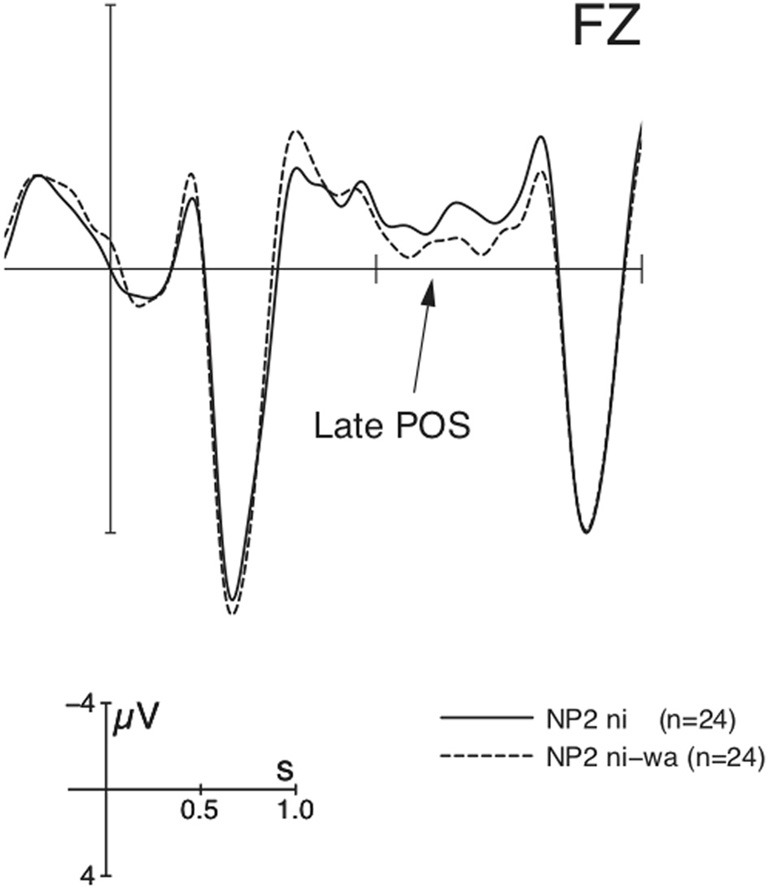
**Grand average ERPs (*n* = 24) time-locked to the dative NP (onset at the vertical bar) at NP2 in Experiment 1 for the comparison of the non-*wa*-marked vs. *wa*-marked dative NPs averaged over all discourse contexts**. Negativity is plotted upwards.

In the 500–700 ms window, there were main effects of CO and NP and an interaction of ROI × CO (with the context effect significant in all ROIs). Pair-wise comparisons between individual contexts revealed reliable differences for each comparison, as presented in Figure 2, i.e., New > Inferred > Given. In addition, there was a ROI × NP × MA interaction in a slightly shorter time-window between 550 and 700 ms. Resolving the interactions further by NP revealed a main effect of MA in both anterior regions only at NP2 but not at NP1. Figure 3 shows that this marker-induced anterior Late Positivity was observed for the *w*a-marked condition vs. non-*wa*-marked condition at NP2.

### Discussion

Experiment 1 showed that discourse context induced a general biphasic N400-Late Positivity pattern. Crucially, the N400 was modulated by sentential position: it was most reduced for a Given NP at the topic position (NP1), and equally reduced for Given and New NPs at the contrastive focus position (NP2). The finding that new NPs can be processed as easily as Given NPs (i.e., Inferred > New/Given at NP2) supports the account of expectation-based processes. Just like expecting Given to be the topic, the new NP does not induce extra cost when it is expected according to its sentential position and preceding context. This finding suggests that the N400 does not reflect processing difference between Given and New *per se* or between topic and contrast, but actually reflects processing differences between unexpected and expected entities. Expectation may arise from context as also shown previously (a Given NP may always be expected, hence the reduced N400 for Given entities) but also from the functional specification of the position, e.g., a contrastive New NP at the sentence-medial position (NP2) is anticipated following the contrastive new context (yielding an N400-reduction relative to the pattern observed sentence-initially).

Crucially, the latter N400-modulation was a position-specific effect. Contrast was not a strong enough cue to reconcile the conflict of new information being at the topic position (NP1). Recall that we used contrastive new NP instead of ordinary new NP in the present study. One could assume that the contrastively new NP at the topic position is justified via the overlap of topic and contrast, i.e., contrastive topic. Nevertheless, we observed a similar pronounced N400 for this contrastively new NP as for the ordinary new NP in the previous study. Therefore, contrastive topic appears to assimilate to the aboutness-topic in the sense that it is also subject to the constraint of givenness (“NP1 = given = topic”). Even though a contrastive NP can be new, it is not expected to appear at the topic position.

As predicted, we also observed a three-way modulation of the Late Positivity (New > Inferred > Given), with the contrastive new NP engendering the most enhanced effect. The contrastive new NP requires the correction of an already established discourse representation structure. The positivity implies that correcting discourse representation structure is more costly than creating new structure. We also observed another (anterior) Late Positivity for *wa*-marked entities at NP2 independent of context. In order to focus on the absent influence of *wa*-marker in the N400 time-window, we reserve further discussion for this anterior Late Positivity to the final discussion.

Our data indicate that the expectation-based parser largely relied on contextual and positional cues but ignored *wa*-marking when generating expectations during topic and contrast processing. At both positions the context-induced N400 was indifferent to whether the dative object was marked by *wa* or not. Given the close correspondence of sentential position and discourse function in Japanese, it is likely that sentential position outranked marker during computation of various cues to generate expectations. Therefore, our findings speak in favor of the theoretical characterization that the positional constraint on topic is independent of marking (e.g., Neeleman et al., 2009; Vermeulen, 2010, 2011). Yet, this leaves us with the question why the language system should make available *wa*-marking at all. If a distinction between a *wa*-marked NP1 and a non-*wa*-marked NP1 exists in the language, a functional explanation should be available. One possibility is that the discourse function of *wa* is beyond just marking a topic or a contrast as examined in Experiment 1. As will be detailed below, *wa*-marked contrastive topic has a particular communicative function that is, it implies that the speaker offers the most informative statement about the topic s/he can make. In this sense, *wa* marks a contrast, which delivers a non-exhaustive listing of all possible alternatives (indicating “at least NP-*wa*”). However, such delicate meaning distinctions are difficult to derive directly from Experiment 1 given that the positional cue was overwhelmingly stronger than the marker. Exhaustivity effects may become more visible when sentential position is controlled and when more markers are compared. Therefore, we conducted Experiment 2 in which we test different markers but keep the critical NPs at the same position, i.e., sentence-initially.

## Exhaustivity (Experiment 2)

Since Experiment 1 revealed a strong impact of sentential position and no effect of marker on discourse functionality, Experiment 2 concentrates on another aspect of discourse functionality, i.e., the implicature derived from the marker independent of position. We thus investigate whether the parser is also sensitive to the subtle differences in implicature transmitted by the marker during contrast processing. The sample stimuli are shown in Table 3, which illustrates that besides the contextual manipulation from Experiment 1, we manipulated the markers of the sentence-initial NP (NP1). NP1 is either a subject marked by nominative marker –*ga*, or an object marked by accusative marker –*o*, or a contrastive topic marked by –*wa*. Crucially, *ga* and *wa* carry additional discourse functional value associated with contrast.

**Table 3:**
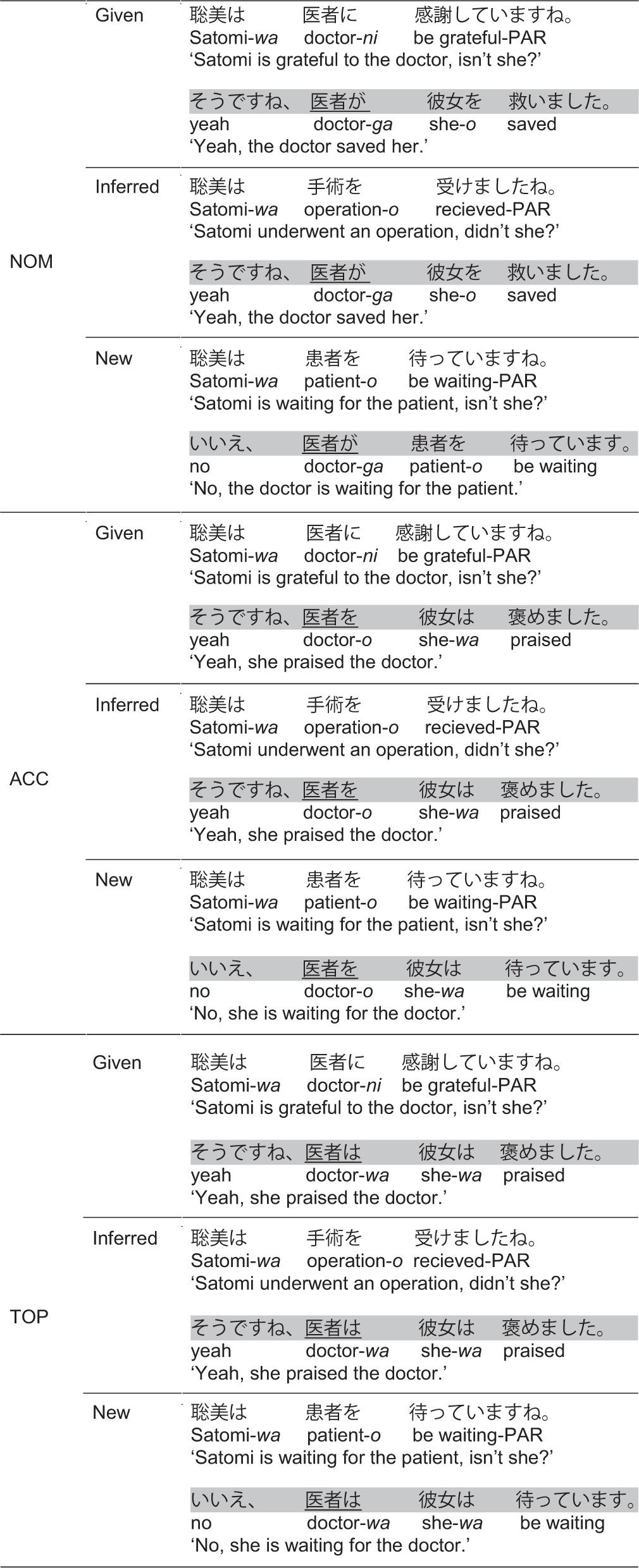
**Examples of critical conditions in Experiment 2 for the factors marker (*ga*; *o*, *wa*), and context (given, inferred, Contrastive New)**.

*Target sentences are shown with gray background and the critical initial NPs are underlined*.

In addition to the well-known fact that *wa* marks topic and contrast in Japanese (Kuno, 1973; Hara, 2006; Tomioka, 2007; Neeleman et al., 2009), Hara (2006) and others argue that contrastive *wa* includes an implicature of uncertainty, which presupposes the existence of a stronger alternative than is asserted [cf. (2c) *Mary-wa passed → Mary and Jane passed*] and implicates that the negation of this alternative may be possible (*It is false that Mary and Jane passed*). Contrastive *wa* (2c) thus generates the implicature that the speaker is uncertain about whether Mary is the only person who passed or whether others passed as well. The speaker signals that this is the most informative statement s/he can utter and indicates that an exhaustive reading is not intended. In contrast, the use of *ga* (2b) clearly indicates an exhaustive reading, signaling that this is the strongest alternative possible (Ono et al., 2000). Accordingly, (2b) (*Mary-ga passed*) implicates *Only Mary passed*. In this sense, the difference between *wa* and *ga* can be characterized by a distinction between “non-exhaustive contrast” and “exhaustive contrast,” which may have consequences for contrast processing.

(2)

- Context: Who passed the exam?
- Mary-ga ukat-ta.
  Mary-NOM pass-Past
  “Mary (and only Mary) passed.”
- Mary-wa ukat-ta.
  Mary-TOP pass-Past
  “(At least) Mary passed.”
  (adopted from Hara, 2006: 19; first discussed in Kuno, 1973: 44)

The crucial question then is whether these marker-specific implicatures affect language processing. Critically, exhaustivity is closely tied to contrastive readings and should therefore only affect the New contrastive contexts in our stimuli set. The Given and Inferred contexts hence serve as control conditions in which exhaustive and non-exhaustive interpretation should not emerge. Accordingly, an interaction of marker and context would support these additional functions.

Example (3) illustrates the three differently marked NPs following the New context. The target sentence starts with a negative particle (“No”), which generates an expectation for a negated target. The negated target can be the verb or an NP, such as the subject, “no, the doctor (not Satomi) is waiting for the patient” (3a), or the direct object, “No, Satomi is waiting for the doctor (not the patient)” (3b).


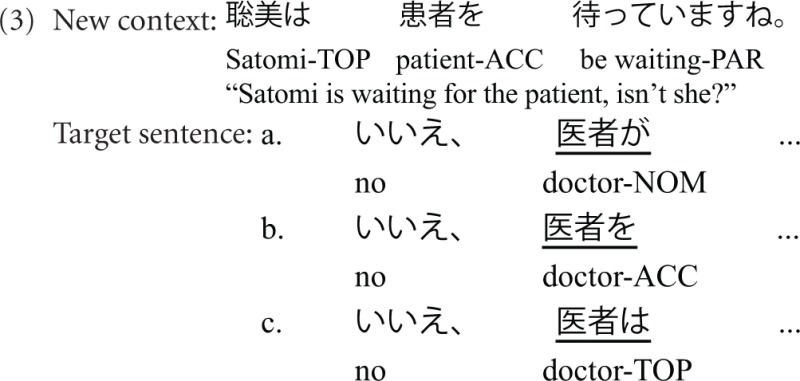


From the perspective of incremental processing, the detection of the negated target is carried out as early as the new NP1 is encountered. The *ga*-marked NP1 is a subject, and represents a contrast to the subject topic of the context sentence. Moreover, the *ga* marker can induce an exhaustively contrastive reading. Example (3a) thus implies that the speaker has a full range of knowledge about who waits for the patient. Among all the people, the doctor and only the doctor is waiting for the patient. In Example (3b), the *o*-marked NP1 is interpreted as an object with its subject topic (*Satomi*) dropped. It is contrastive not because *o* is a contrastive marker. Rather it receives contrastive reading from the context via forming a parallel structure with the context sentence (i.e., NP-*wa* NP-*o* structure though the NP-*wa* is dropped). According to parallel structure and function assignment (cf. Gordon and Scearce, 1995; Streb et al., 1999), the *wa*-marked NP1 in (3c) is preferred to be analyzed as a subject parallel to the subject topic in the discourse context, like (3a) (see also Wolff et al., 2007 for subject-preference associated with an ambiguously case-marked NP, i.e., *wa*-marked NP). Unlike the *ga* marker, neither *wa* nor *o* can induce an exhaustive contrast reading.

The different markers at NP1 render it possible to examine whether and how discourse markers interact with context-induced expectations during topic and contrast processing by specifying the discourse function of the contrastive marker in a more delicate way. *Ga* vs. *wa*/*o* offers a comparison within contrast i.e., a contrast with or without exhaustive listing. In the Given and Inferred conditions where the target sentence starts with *yeah*, the parser generates an expectation for a given NP to be the topic. Under these contexts, the *wa*-marked NP is a topic without contrast. Also the *ga*-marked NP1 can receive a neutral reading [“descriptive *ga*” as shown (1c)] without a contrastive reading. Since previous findings from Japanese revealed that *wa/ga* alternation does not influence topic processing (Hirotani and Schumacher, 2011), we thus focus on contrast processing (i.e., the contrastive New context), taking the other two contexts as control conditions.

On the basis of the findings from Experiment 1, we should again observe a context-induced biphasic N400-Late Positivity pattern at the critical position of NP1 (New > Inferred > Given). However, unlike Experiment 1, we expect a stronger influence of discourse marker yielding an interaction of context and marker, reflected by an N400-modulation in the contrastive New context, but not in the Given and Inferred contexts. If exhaustivity matters, we should observe a processing difference between NP1-*ga* and NP1-*wa/*NP1-*o*. Alternatively, if these markers do not amount to influence contrast processing, we should only observe the context-induced N400 pattern but no interaction of context and marker in the N400 time-window.

### Methods

#### Participants

Twenty-six monolingually raised native speakers of Japanese participated in the experiment after giving informed consent (18 women; mean age: 25.1 years; range: 18–38 years). None had participated in Experiment 1. Four participants were excluded from data analysis due to excessive EEG artifacts and high error rates in the behavioral task.

#### Materials and procedure

We constructed 40 trials per condition as shown in Table 3. This resulted in 360 critical trials in total. The experimental procedure, task, and EEG recording parameters were identical to Experiment 1 with the exception that the experiment was conducted as one session with eight blocks.

#### Data analysis

The ERP data were analyzed in analogy with Experiment 1, except that ANOVAs were computed with the factors discourse context (CO—Given, Inferred, New) and marker (MA—*ga*, *o*, *wa*), as well as the topographical factor ROI.

### Results

**Figure 4:**
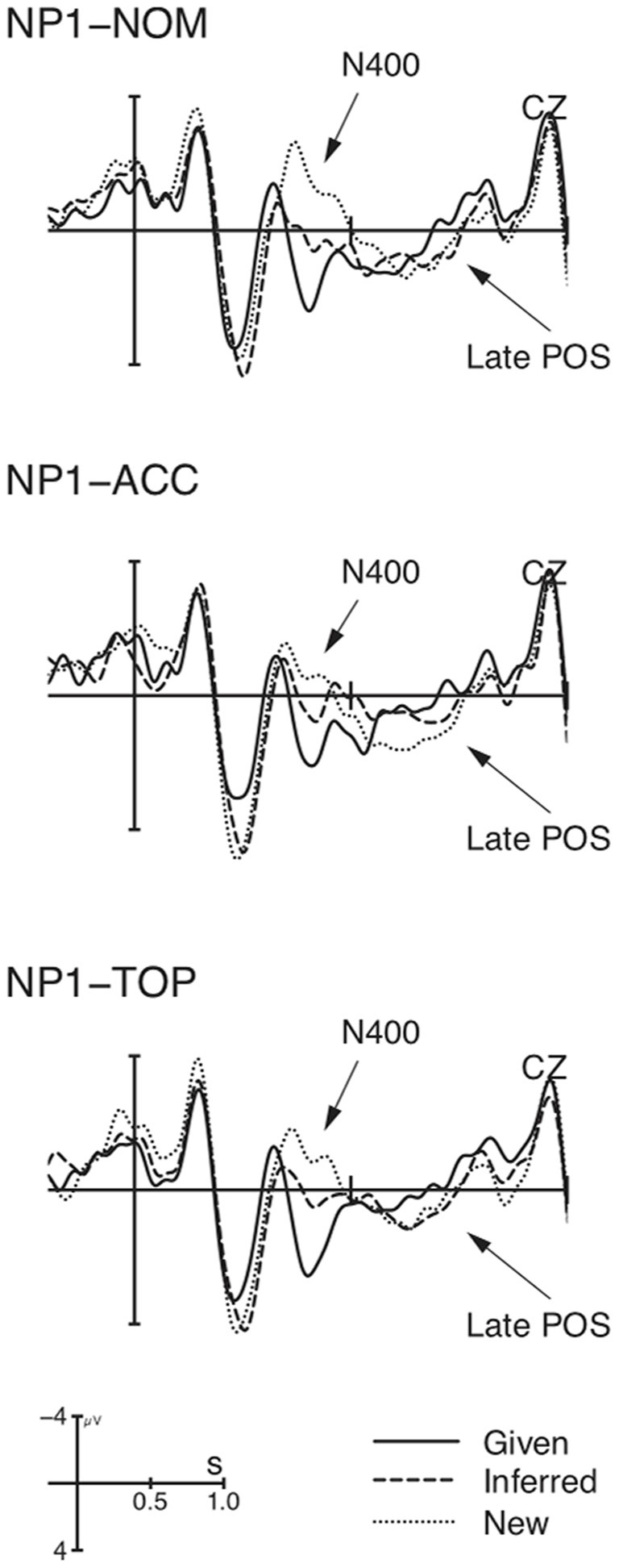
**Grand average ERPs (*n* = 22) time-locked to NP1 in Experiment 2 (onset at the vertical bar) in the Given, Inferred, Contrastive New contexts**. Negativity is plotted upwards.

**Figure 5:**
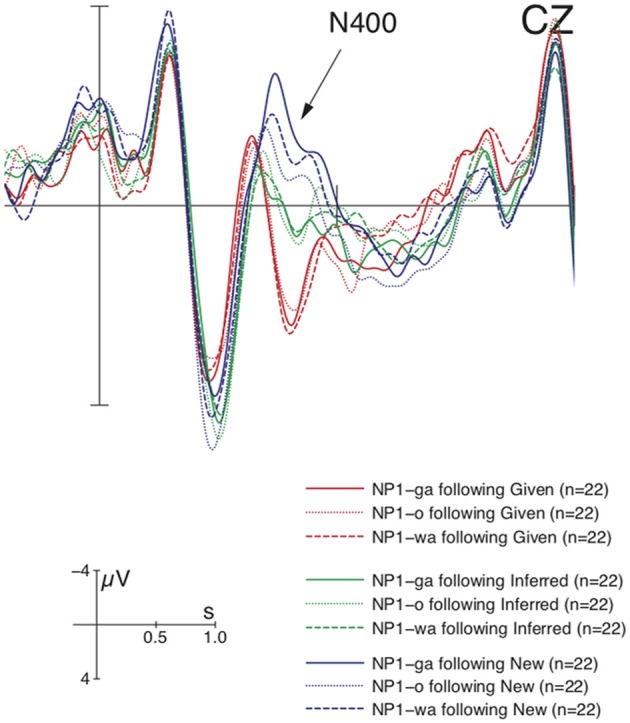
**Grand average ERPs (*n* = 22) time-locked to NP1 in Experiment 2 (onset at the vertical bar) for the comparison of *ga* vs. *o* vs. *wa* in the Given, Inferred, Contrastive New contexts**. Only the NP1 in the new context engendered a marker-modulated N400 in comparison with the other two contexts. Negativity is plotted upwards.

Figure 4 shows that the ERP-responses time-locked to NP1-onset engendered the basic data pattern of context manipulation as in previous studies, i.e., a graded N400 for New, Inferred, and Given, followed by a Late Positivity for New/Inferred vs. Given. More importantly, the context-induced N400 was modulated by marker, as shown in Figure 5.

This observation was supported by statistical analysis (see Table 4). In the N400-window (350–500 ms), there was a main effect of CO, reflected by significant differences between pair-wise comparisons of individual contexts. Furthermore, there was an interaction of CO × MA, which resulted from a significant difference in the comparison of *ga* vs. *o* in the New context. Whereas visual inspection suggested the *wa*-marked NP1 showed an intermediate N400 between the *ga*-marked NP and *o*-marked NP, the comparisons of *o* vs. *wa*, and *g*a vs. *wa* did not reach a significant difference in this context. In the Late Positivity time-window (600–800 ms), there was a main effect of CO, yielding significant pair-wise differences between New and Given, and between Inferred and Given, but not between New and Inferred contexts^5^. In sum, Experiment 2 revealed a general context-induced N400-Late Positivity pattern. Crucially, it showed that the N400 can be modulated by discourse marker.

**Table 4:** **Analysis of variance (ANOVAs) of the mean ERP amplitudes in Experiment 2**.

**N400 (350–500 ms)**					
			***df***	***F***	***p***
**LATERAL REGIONS**					
CO			2.42	51.06	^***^
		Inferred vs. Given	1.21	35.08	^***^
		New vs. Given	1.21	88.94	^***^
		New vs. Inferred	1.21	22.63	^***^
MA × CO			4.84	2.89	^*^
*ga* vs. *o*	CO	New	1.21	13.84	^**^
**MIDLINE REGIONS**					
CO			2.42	63.90	^***^
		Inferred vs. Given	1.21	40.47	^***^
		New vs. Given	1.21	98.02	^***^
		New vs. Inferred	1.21	35.97	^***^
ROI × CO			10.210	4.65	^**^
all ROIs	CO		1.21	>41.90	^***^
MA × CO			4.84	2.62	^*^
*ga* vs. *o*	CO	New	1.21	11.51	^**^
**Late Positivity (600–800 ms)**					
**LATERAL REGIONS**					
CO			2.42	17.17	^***^
		Inferred vs. Given	1.21	30.11	^***^
		New vs. Given	1.21	21.48	^***^
ROI × CO			6.126	5.50	^***^
all ROIs	CO		2.42	>10.44	^***^
**MIDLINE REGIONS**					
CO			2.42	12.99	^***^
		Inferred vs. Given	1.21	18.72	^***^
		New vs. Given	1.21	18.09	^***^

Note:

* p < 0.05;

** p < 0.01;

*** *p < 0.001*.

### Discussion

Experiment 2 replicated the findings of Experiment 1 with respect to the context-induced N400-Late Positivity pattern at the sentence-initial position. More importantly, Experiment 2 revealed a more fine-grained picture of contrast processing during which the context-induced N400 changes as a function of the NP's marker following the New context. It is worthy to note that the marker-modulated N400 cannot merely reflect a difference in processing different forms of markers or grammatical function analysis, because if this was the case we should have observed a similar N400 pattern in the Given and Inferred contexts as well. For the same reason, a topic-shift account can also not explain our data (cf. Hung and Schumacher, 2012 for N400-effects for topic-shift). Therefore, we attribute this N400-modulation to the subtle differences in contrastive meaning. Experiment 2 starts from the assumption that the discourse marker may convey a unique function which the sentential position cannot. We found the main difference was attested between the two most distinct markers, *ga* vs. *o*, which suggests that a marker-induced exhaustive contrast can influence the early processing of a new NP. It should be emphasized that the exhaustivity-modulated N400 can only be observed during contrast processing, since no enhanced N400 was observed during topic processing where *ga* just marks new information without contrast (a descriptive *ga*) in the present experiment. This is also evident from the previous study by Hirotani and Schumacher (2011), in which the authors used a new context without contrast and did not observe N400-differences for *ga* vs. *wa* (a descriptive *ga* again). Given that both *ga*-marked NP1 and *o*-marked NP1 are contrastive by virtue of context, the enhanced N400 for *ga* should be exclusively due to the additional cost of deriving the exhaustive reading from this marker. The additional implicature of an exhaustive reading is not expected.

However, because differences between the *wa* marker and the other two markers were not significant, the exact status of *wa* is worth considering. The processing behavior of the initial *wa* may in fact reflect the complicated nature of contrastive topic. As argued in the theoretical literature, topic and contrast do not exclude each other but rather represent different layers in information structure (Büring, 2007; Krifka, 2008). The processing behavior of the initial *wa* substantiates a dynamic view of discourse processing: during on-line processing, the parser first meets the structural need of establishing a topic (contrastive or not)^6^. To derive the contrastive meaning is rather a secondary task.

Furthermore, unlike Experiment 1, the Late Positivity in Experiment 2 was more enhanced for New and Inferred vs. Given NPs. The absence of the three-way modulation is most likely confounded by the N400-difference in the contrastive New context. As is evident from Figure 4, the accusative marked NP registered a three-way modulation, but the other two markers that exerted more pronounced N400-amplitudes did not.

Extending the findings from Experiment 1, Experiment 2 showed an interaction of context and marker caused by the exhaustive contrast. During contrast processing, there is an expectation for a non-exhaustive reading, and evoking an extra exhaustive reading (*ga* marker) exerts costs.

## General discussion

The findings of Experiments 1 and 2 can be summarized as in Table 5. We found a strong impact of context but also effects of sentential position and marker on referential processing. Position served as a cue for the generation of discourse functional expectations. Experiment 1 revealed that new NPs in contrastive focus position (NP2) are facilitated following a contrastive context. Markers also demonstrated sensitivity to contrastive context when they carried additional exhaustivity features. In this case, the strongest possible answer (exhaustive contrast) exerted costs.

**Table 5:** **ERP components and patterns at the critical NPs**.

	**Position**	**N400**	**Late Positivity**
Experiment 1	NP1	New > Inferred > Given	New > Inferred > Given
	NP2	Inferred > New/Given	1. New > Inferred > Given
			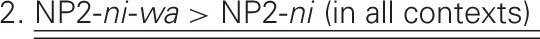
Experiment 2	NP1	1. New > Inferred > Given	New/Inferred > Given
		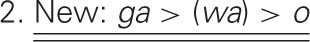	

*“>” means the left engendered a more pronounced effect as opposed to the right. Single underlines and double lines highlight the effects of sentential position and marker, respectively. Others are context-induced effects*.

The general pattern supports the two-stage structure argued for in the SDM: *Discourse Linking*—indexed by the N400—computes various cues to reflect the expectation of an upcoming referent and initial linking attempts with discourse, while *Discourse Updating*—indexed by the Late Positivity—reflects discourse-internal reorganization and integration. Expectation-based parsing must be temporally dissociated from the assessment of discourse representation structure. The former process is guided by contextual requirements for a global given-new distinction but also specific demands of information processing as reflected by the subtle differences arising from the interaction of the contrastive context with sentential position and discourse marker. This process results in the updating of discourse structure. Below, we address the contribution of the current findings to understanding the nature of the N400 and the Late Positivity, comparing the functional interpretation of these two components in classic psycholinguistic literature. Finally, we discuss the implications of our findings for models of language processing.

### N400 and information structural expectation

The most important finding in Experiment 1 is the observation of a reduced N400 for new vs. given when the new NP is expected to be the contrastive focus in accord with contextual and positional cues. This finding from contrast processing, together with those from topic processing, provides strong evidence in support of an expectation-based mechanism. The data clearly indicate that there are no absolute processing demands for new vs. given, rather, costs arise from information structural unexpectedness: when a new NP is not expected (topic), linking costs accrue; when a new NP is expected (contrastive focus), no linking costs result. Furthermore, the present study revealed that the expectation is generated from various cues at different linguistic levels (not restricted to lexical cues). Therefore, our finding supports an expectation-based account nourished by discourse-functionality over a pure lexical activation account for the N400 (see Kutas and Federmeier, 2011 for an overview of lexical N400, and see Brown and Hagoort, 1993; Hagoort et al., 2009 for post-lexical integration N400). This is also evident from Experiment 2, in which extra-lexical information from markers such as the marker-induced contrastive reading (i.e., an exhaustive list reading) also affects the neural processes of lexically identical NPs. This finding provides further evidence for the claim that the N400 is sensitive to discourse-level information as previously argued for referential processing of prosodically encoded information status (on lexically identical target words; Schumacher and Baumann, 2010) and responses to topic and non-topic questions (again, measured on lexically identical target words, Hung and Schumacher, 2012).

Moreover, in contrast to the off-line results of the acceptability rating of the sentences used in Experiment 1 (reported in the Appendix), which showed an equally strong influence of sentential position and markedness, the on-line processing results suggest that the parser generates expectations for an upcoming referential expression on the basis of computing all available cues but not in an equal way. For example, discourse context has an overwhelming power, outranking sentential position, which outranks *wa* marker in guiding the expectation-based processes (Experiment 1). In fact, a stronger influence of sentential position over *wa* marker is not surprising. This is compatible with observation of mapping topic and contrastive focus onto discrete positions independent of marker. We assume that the interaction of discourse context and sentential position can be observed widely across languages, as in many languages sentential position is a reliable cue and sometimes even the only available cue to encode information structural distinction (see LaPolla, 1995). However, this does not mean that the parser ignores discourse functions of the marker at all. In fact, when the sentential position is controlled for, the marker has an early impact on the context-induced contrast (Experiment 2). The additional contribution of exhaustive listing readings to contrast might thus also explain why definiteness marking had no effect on early processing stages in German (Schumacher, 2009). This allows for the predictions that inducing specificity may pattern with exhaustive listing interpretations in that it reduces the alternative set to a uniquely identifiable referent.

Taken together, the present study revealed that the new referent is not costly when the respective entity is expected (contrastively focused). Additional meaning aspects (e.g., exhaustivity) may exert costs during early processing stages. Overall, the expectation-based parser draws on various cues that carry discourse functions and it selectively uses cues by computing which cue is more informative during topic and contrast processing. The discourse-dependent N400 observed here is therefore more compatible with a view that considers the discourse functional contribution of different cues, rather than a strict lexical view.

### Late positivity and discourse updating

The context-induced Late Positivity observed for the contrastive new referent (Experiment 1) can be interpreted in a straightforward manner. An expected new referent (contrastive focus) renders reduced linking demands, however, it engenders increased costs during the construction of discourse representation structure (i.e., *Discourse Updating*). This is because the new referent requires the correction (after negation) of previously introduced discourse structures in addition to the creation of a new discourse unit.

In addition, an anterior positivity was observed in Experiment 1 as well, which we discuss now. The second *wa*-marked NP registered an anterior Late Positivity independent of context. The anterior distribution is different from those “standard” Late Positivity effects that have been reported for discourse processing so far. We propose that this effect can be best captured in terms of “discourse complexity” (Kaan and Swaab, 2003). Kaan and Swaab (2003) observed a similar anterior effect for contexts containing two potential referents for agreement (e.g., *I cut the cake beside the pizzas that were brought by Jill*.) vs. one referent (*The man in the restaurant doesn't like the hamburgers that are on his plate*.) relative to the verb; they associated this effect with increasing discourse complexity and the need for ambiguity resolution. This explanation seems to be most compatible with the present data. The second *wa*-marked NP introduces a second salient entity and consequently leads to complexity at the discourse level, i.e., the contrastive focus (the *wa*-marked NP2) and the topic (the *wa*-marked NP1) compete for discourse salience. This explanation is also compatible with the anterior Positivity observed for a single *wa*-marked NP in the previous Japanese study (Hirotani and Schumacher, 2011), where the *wa*-marked NP elicited an anterior Late Positivity as opposed to the *ga*-marked referent in discourse contexts that did not support an outright topic reading of the NP (i.e., the *wa*-marked subject in the Inferred context). Recent findings form Japanese also support a connection between the Late Positivity and discourse complexity. When the referent is not straightforwardly recoverable, i.e., when more than one NP compete for a dropped argument, a similar positivity results (Wolff et al., 2008; Wolff, 2009). All these findings provide good evidence to suggest that the Late Positivity correlates with the referential realization of an NP in a discourse. The data thus seem to suggest that a topic-shift (Hirotani and Schumacher, 2011) or a referent competing in salience with the topic of a sentence (i.e., a second *wa*-marked NP in Experiment 1; see Cowles et al., 2007 for contrastive focus and topic with the same salience) will increase the discourse complexity and call for discourse-internal reorganization. However, as the anterior Late Positivity did not consistently interact with the discourse context (considering that it was context-dependent in Hirotani and Schumacher, 2011, but not in Experiment 1), the exact circumstances under which it occurs need to be further examined in future research.

How do these Late Positivities compare with other late positive deflections found during language comprehension? The literature discusses numerous instances of “semantic P600s” evoked during argument processing at the sentence-level (e.g., *The fox that hunted the poachers)*, which typically results from syntax-semantics mismatches (see Bornkessel-Schlesewsky and Schlesewsky, 2008 for an overview), or during other combinatorial operations resulting from a type mismatch (e.g., *The waitress said that the ham sandwich wanted to pay*; Schumacher, 2011, in press). These findings could be accounted for in a unified manner when assuming a domain general updating mechanism. In this vein, discourse representational modifications must be carried out when information in the input is conflicting. Incrementally built representations are corrected (in the case of semantic reversal anomalies; *fox* vs. *poachers*) or modified/enriched (in the case of *ham sandwich* metonymy and other types of non-literal meaning composition) to obtain a felicitous representation. Within the SDM, these operations can be considered instances of information packaging, where the reorganization or “unpacking” of information structure exerts updating demands.

### Implications for the processing architecture

So far, we have discussed the functional significance of the discourse-related N400 and Late Positivity separately. However, what are the implications of the biphasic pattern for the language architecture?

The traditional association of N400 with semantic processing and P600 with syntactic processing has for instance been challenged by findings associated with the semantic P600. In order to derive the presence of the (semantic) P600 with a concurrent absence of the N400, different models have provided different accounts (see Brouwer et al., 2012 for a recent overview). Multi-stream models account for the two neural responses under the assumption that there are two parallel processing streams, a semantic/plausibility-based processing stream and a syntactic/algorithmic processing stream, and either of the two responses occurs resulting from a mismatch or competition between these two streams (e.g., Kim and Osterhout, 2005; van Herten et al., 2006). However, Brouwer and colleagues pointed out that all multi-stream models (except the eADM in Bornkessel-Schlesewsky and Schlesewsky, 2008) have problems in deriving the concurrent occurrence of the N400 and the Positivity, which is also observed by the biphasic N400-Late Positivity pattern in our study. In addition, Brouwer and colleagues observe that the multi-stream models share an integration view of the N400. In response, Brouwer et al. propose a single-stream model, MRC (*mental representation of what is being communicated*), which treats N400 as an index of lexical retrieval while the P600 reflects difficulties in integrating information into the discourse representation. In this vein, all positivities—including semantically and syntactically derived effects—are accounted for in a unified manner, because thematic and syntactic reanalysis and revision are considered to require updating of the discourse representation. This explanation is in line with the proposal of the SDM that the Late Positivity represents a domain general updating mechanism (see also Hirotani and Schumacher, 2011; Schumacher, 2011).

The SDM and the MRC differ with respect to the functional interpretation of the N400. While the MRC considers the N400 to reflect lexical retrieval from long-term memory, the SDM endorses an expectation-based view on the basis of effects from bottom-up and top-down information. A lexical view of the N400 is challenged by the findings that the N400 is modulated by sentential position and marker at lexically identical words. The present data thus demonstrate that the expectation-based parser makes use of cues from various sources. Note however, that the MRC seems to allow for a certain degree of top-down influences as well (“top-down information […] adds to the activation pattern […]; it does not constrain the pattern of activation”, p.134). Another framework, which holds the integration view, is the MUC (*memory, unification, control* in Hagoort et al., 2009) with the central notion of semantic unification. However, the N400 in semantic unification includes integration and enriched composition, while the SDM functionally dissociates expectation-based parsing on the one hand and discourse-internal operations—including certain types of enrichment—on the other hand.

Taken together, within the SDM, the N400 is interpreted to reflect expectation-based parsing based on the computation of various cues and the Late Positivity to reflect discourse updating, resulting from discourse integration and assessment of discourse representation structure. These functional interpretations are partly compatible with other models such as MRC and MUC. However, neither of the existing models is sufficient to explain the biphasic N400-Late Positivity observed in the present investigation. We therefore advocate the SDM as a framework that can account for specific instantiations of referential processing. Finally but critically, the SDM can also predict an independent occurrence of an N400 or a Late Positivity, because *Discourse Linking* and *Discourse Updating* are considered as two independent processes within the SDM. This is evident from the fact that the N400 and the Late Positivity do not necessarily occur together and that demands reflected in N400 differences are not necessarily mirrored by demands in the Late Positivity and vice versa (Burkhardt, 2007; Schumacher, 2009; Schumacher and Hung, 2012). Since we have focused on referential processing in the present and previous research, the architecture appears to be single-streamed. However, our view of the Late Positivity is couched within the proposal that updating mechanisms are triggered by conflicting information [e.g., an expression can be linked to an anchor, but when discourse representation is assessed it turns out that there is no corresponding discourse unit yet and the system resolves this conflict by creating an independent discourse referent (see our inferred conditions); a type mismatch occurs between a predicate and its argument (see *the ham sandwich wanted to pay* cases in Schumacher, 2011)] and as such the SDM joins the multi-stream models with the unique feature that the final processing phase reflects revision and updating at the level of discourse representation.

## Conclusion

The present study examined the online processing of topic and contrast assigned by cues such as discourse context, sentential position, and marker during referential processing in Japanese. Our results support an expectation-based parser, which is subject to the competition between multiple cues, by showing a reduced N400 for an expected new NP (contrastively focused). The cost of processing a new NP rather occurs in the later process of discourse updating, in which the new NP's occurrence requires updating and correcting of discourse representation built so far, which is indexed by an enhanced Late Positivity.

### Conflict of interest statement

The authors declare that the research was conducted in the absence of any commercial or financial relationships that could be construed as a potential conflict of interest.

## Footnotes

^1^Studies on this line have focused on rather diverse topics, depending on individual definitions of what a “context” is (cf. Schumacher, 2012 for a review). On the one hand, context in a wide sense includes world and event knowledge, specific information about the interlocutors' knowledge, belief, and interests etc., which have been investigated as cues for the formation of expectations for upcoming words (e.g., van Berkum et al., 2008, 2009; Hagoort et al., 2009). On the other hand, traditional studies on word processing use context in a narrow sense, where it is restricted to sentence-level information retrieved in an incremental manner, i.e., a sentence unfolds incrementally and the words preceding the target word represent the context (sentence context, e.g., van Petten and Kutas, 1990; van den Brink et al., 2001).

^2^Prosody is another cross-lingusitically important means to convey information structural notions; givenness has prosodic reflexes in numerous languages of the world and so has focus (Lambrecht, 1994; Cruttenden, 2006; Büring, 2007; Krifka, 2008). In Japanese topical *wa* and contrastive *wa* can be distinguished on the basis of different prosodic patterns: compared to the topical *wa*, there is a significant drop in fundamental frequency (*F*_0_) contour immediately after the contrastive *wa* (Nakanishi, 2001). Note however that we presented stimuli visually.

^3^Tomioka (2007) assumes contrastive topic status for the sentence-medial entities marked by *wa*; but see Neeleman et al. (2009) for a refute.

^4^According to Miyagawa (1987, 1989), the presence and absence of case/postposition is directly related to the interpretation of an NP's discourse function: case markers like −*ga* (for subject) and −*o* (for object) are omitted when the wa marker is used, and in this case, −*wa* can be topical or contrastive. By contrast, postpositions like kara (“from”), made (“until”), de (“at”), and so on must be overtly present in combination with the wa marker, and in this case, −*wa* should be exclusively interpreted in a contrastive manner. However, −*ni* can be used as a case marker or a postposition. Therefore, one could argue that *wa*-marked dative objects with an omitted case particle (NP-*wa*) would align with topic reading, while overtly realized dative objects (NP-*ni*−*wa*) would align with contrastive reading. However, such a one-to-one mapping is not warranted given the analyses reviewed above. The acceptability judgment conducted by Nagata (1994) showed that *ni*-omission depends on how easy Japanese readers can recover −*ni* with the help of verb meaning, sentence context, or world knowledge. The exact interpretation of the *wa*-marked dative object thus depends on multiple factors including sentential position and discourse context. This is also consistent with feedback from five Japanese informants in a sentence completion task involving sentences beginning with NP-*ni*−*wa*. Therefore, we do not think that the contrastive reading of an NP is induced from the combination of −*ni*-*wa* itself in the present study.

^5^The absence of a difference between New and Inferred (in contrast to Experiment 1) may be due to spill-over effects from the N400 window. See discussion below.

^6^Two pieces of evidence speak for the claim that topic is prior to contrast. First, Vermeulen (2010) argues that a contrastive topic—like aboutness-topic—is under the positional constraint for a topic. Second, reanalysis effects are observed at NP2 in Experiment 2: we obtained a statistically significant N400 (290–340 ms) for the condition of NP1-*o* NP2-*wa* as well as the condition of NP1-*wa* NP2-*wa* in comparison with the condition of NP1-*ga* NP2-*o* (all Fs > 6.20, *p* < 0.03). The N400 for the *wa*-marked NP2 after the *o*-marked NP1 can be explained by the fact that a second topic is not expected, since at NP1 a topic-drop reading is adopted. In a similar way, the N400 for the *wa*-marked NP2 after the *wa*-marked NP1 suggested that a second topic is not expected given the initial *wa*-marked NP1 is already analyzed as topic. In fact, this is also observed in Experiment 1 where we found a similar reanalysis N400 for NP2-*ni*−wa vs. NP2-*ni* following NP1, a pronominal topic.

## Appendix

### Pre-test of experiment 1

The acceptability rating study served to provide us with a first indication of whether there is an interaction between discourse context, NP's sentential position and marker. In addition, we wanted to determine the best marking of the subject NPs in the experimental stimuli. Previous studies have shown that the parser in Japanese is more sensitive to case information than to the position of an NP (Yamashita, 1997). Under a discourse environment, we assume that the parser is sensitive to discourse marker. Since Japanese allows two subsequent *wa*-marked NPs, this may increase the processing difficulty (cf. Bever, 1974; Kuno, 1974). For example, the sequence of “NP_S_-*wa* NP_O_-*ni*-*wa*…” (subscripts indicate the grammatical function) leads to a less preferred and infrequent double *wa*-construction. In order to facilitate processing, one would probably adopt a strategy to maximally distinguish markers by using *ga* instead of *wa* for the subject NP. This would mean that in some conditions “NP_S_-*wa* NP_O_-*ni*-*wa*…” does sound less natural than NP_S_-*ga* NP_O_-*ni*-*wa*…” (the same holds for “NP_O_-*ni*-*wa* NP_S_-*wa* …” vs. “NP_O_-*ni*-*wa* NP_S_-*ga* …”). Since we wanted to use either *wa* or *ga* to mark the subject for all the conditions under investigation, the pre-test was also intended to identify the most naturally sounding subject marker (*wa* or *ga*) for our conditions.

We manipulated the dative object's marker as well as the subject's marker, as shown in (i), following each of the three discourse contexts and in two word order variations. This resulted in a 2 (S-*ga* vs. S-*wa*) × 2 (O-*ni* vs. O-*ni*-*wa*) × 2 (subject-initial vs. object-initial) × 3 (Given vs. Inferred vs. New) design with 24 conditions in total. If *ga*–*wa* variation at the subject plays a role in the sentence's naturalness, we should see a significant difference between all the *wa*-marked subject conditions and all the *ga*-marked subject conditions in the acceptability of the sentences.

#### Participants

Twenty-three native speakers of Japanese (16 women) took part in the questionnaire study. Eighteen of them were students of the University of Mainz, who had only been in Germany for approximately one month, and five native speakers of Japanese were residing in Japan. They were between 19 and 54 years old (mean age: 25.4 years).

#### Materials

Considering the large number of conditions, three passages were selected for each condition used in Experiment 1. The resulting 72 critical sentences were randomly interspersed with 28 filler sentences. In order to display varying degrees of acceptability, the fillers were comprised of the most natural and not at all natural sentences with intransitive and transitive verbs.

#### Procedure

Participants judged the acceptability of two-sentence passages on a 4-point scale (1 = “natural”; 4 = “not at all natural”). They were required to judge the passages based on the criteria of whether the target sentence forms a natural continuation after the context sentence according to their intuition.

#### Results

The mean acceptability ratings obtained for the materials of the present experiment are shown in Table A1. In general, the sentences with *wa*-marked subject were significantly more acceptable than those with *ga*-marked subject [*F*_1(1, 22)_ = 23.96, *p* < 0.0001]. This observation is also confirmed by individual comparisons between *wa*-marked subject and *ga*-marked subject in each condition. Only four conditions were judged more acceptable when the subject was marked by *ga* rather than by *wa* (these are underlined in Table A1) and only one of them reached a significant difference (highlighted by double lines in Table A1), i.e., the condition in which the *wa*-marked, given dative object is followed by a *wa*-marked subject (NP_O_-*ni-wa* NP_S_-*wa*). This could be due to a conflict in salience rankings: while the subject topic is more salient than the dative one, it occupies a less salient position in a sentence. On the basis of these findings, we chose the *wa*-marker for all subject NPs in Experiment 1, and report further statistical analyses for the pretest within the 12 *wa*-marked subject conditions only.

**Table A1:** **Mean acceptability ratings for the critical conditions**.

			***S-wa***	***S-ga***
NP1	*O-ni*	Given	1.81 (0.93)	2.22 (0.70)
		Inferred	*2.25 (0.76)*	*2.22 (0.59)*
		New	2.48 (0.80)	2.55 (0.80)
	*O-ni-wa*	Given		
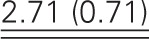
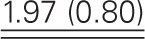
		Inferred	2.07 (0.75)	2.59 (0.79)
		New	*3.32 (0.83)*	*3.06 (0.70)*
NP2	*O-ni*	Given	1.90 (0.73)	2.75 (0.71)
		Inferred	*2.14 (0.62)*	*2.09 (0.66)*
		New	1.72 (0.80)	2.87 (0.88)
	*O-ni-wa*	Given	2.30 (0.80)	2.81(0.67)
		Inferred	2.67 (0.97)	3.07 (0.70)
		New	2.58 (1.01)	3.09 (0.77)
Mean			2.33 (0.53)	2.60 (0.56)

*Wa vs. ga are the marker of the subject, and ni vs. ni-wa are the marker of the dative object. Standard deviations are given in parentheses. 1 = “natural”; 4 = “not at all natural.” Comparison in each condition showed wa-marked subjects were judged more acceptable than ga-marked subjects except four conditions (highlighted by a single line). Only one out of four conditions reached a significant difference (highlighted by double lines)*.

Repeated measures analysis of variance (ANOVA) were computed for the acceptability ratings involving the within-participants factor discourse context (CO), NP's sentential position (NP), discourse marker of dative object (MA), and the random factor participants (*F*_1_). For the reason that we selected different items for each condition to avoid lexical repetition, the random factor item (*F*_2_) is not applicable in the acceptability study.

The statistical analysis revealed main effects of CO, *F*_1(2, 44)_ = 4.49, *p* < 0.02, NP, *F*_1(1, 22)_ = 18.21, *p* < 0.001, and MA, *F*_1(1, 22)_ = 18.04, *p* < 0.001. Furthermore, there was an interaction of NP × CO, *F*_1(2, 44)_ = 27.22, *p* < 0.0001, as well as MA × CO, *F*_1(2, 44)_ = 9.92, *p* < 0.001. Subsequent pair-wise comparisons between individual discourse contexts after resolving the interaction of NP × CO showed that Inferred and Given differed significantly from one another at NP2, *F*_1(1, 22)_ = 7.42, *p* < 0.02, while both of them differed significantly from New at NP1, New vs. Given, *F*_1(1, 22)_ = 17.1, *p* < 0.001, and New vs. Inferred, *F*_1(1, 22)_ = 44.6, *p* < 0.0001. Resolving the interaction of MA × CO by MA showed that Given differed from Inferred when the dative object was not marked by *wa*, *F*_1(1, 22)_ = 10.31, *p* < 0.005. However, both Given and Inferred differed significantly from New when the dative object was marked by *wa*, New vs. Given, *F*_1(1, 22)_ = 6.63, *p* < 0.02, New vs. Inferred, *F*_1(1, 22)_ = 8.55, *p* < 0.01.

#### Discussion

The acceptability ratings revealed that sentences with *wa*-marked subject NPs were judged more acceptable than the sentences with *ga*-marked subject NPs in general. We thus used *wa*-marked subjects for all the conditions in Experiment 1. More importantly, within the selected 12 *w*a-marked subject conditions, the acceptability rating results showed that there is a clear interaction of discourse context and the NP's sentential position as well as *wa* marker in the acceptability of the different sentence pairs. However, as the NP's sentential position and marker appear to be equally strong in influencing the acceptability rating, which could be due to the fact that the off-line acceptability rating study reflects the final outcome of the interaction among multiple factors, i.e., the final outcome after reading the whole sentence, it is essential to obtain online measures in order to investigate to what extent these factors influence local processing decision during reading the sentences.
